# Neutrophil to lymphocytes ratio in deep infiltrating endometriosis as a new toll for clinical management

**DOI:** 10.1038/s41598-024-58115-6

**Published:** 2024-03-30

**Authors:** Mattia Dominoni, Marianna Francesca Pasquali, Valentina Musacchi, Annalisa De Silvestri, Matteo Mauri, Virginia Valeria Ferretti, Barbara Gardella

**Affiliations:** 1 Department of Clinical, Surgical, Diagnostic and Pediatric Sciences, University of Pavia, 27100 Pavia, Italy; 2 Department of Obstetrics and Gynecology, IRCCS Fondazione Policlinico San Matteo, 27100 Pavia, Italy; 3 SSD Biostatistica e Clinical Trial Center, IRCCS Fondazione Policlinico San Matteo, 27100 Pavia, Italy

**Keywords:** CA 125, Endometriosis, Neutrophil, Lymphocytes, NLR, Immunology, Biomarkers, Molecular medicine, Pathogenesis, Risk factors

## Abstract

Several mechanisms, including altered local and systemic immune system, apoptosis, and new angiogenesis, are responsible for the development and progression of endometriosis. Over the years many markers have been studied, like CA 125 and, recently, neutrophil-to-lymphocyte ratio (NLR). This tool is cost-effectiveness and non-invasiveness as a marker of systemic inflammatory diseases. The aim of this study is to assess the role of NLR in the real-life management of patients with endometriosis in order to evaluate the possible association between this value and symptoms. We performed a retrospective analysis of 199 premenopausal women affected by endometriosis, from January 2013 to December 2020, evaluating the characteristics of disease, the symptoms and the NLR. Analyzing the neutrophiles, the mean ± SD value was 6.1 ± 4.5 × 10^3^/ul, while for lymphocytes mean ± SD value was 1.8 ± 0.7.NLR was categorized according to its median value (> 2.62 vs ≤ 2.62). The comparison between NLR values and CA 125, endometriosis stage, dysmenorrhea and presence of chronic pelvic pain, adjusting for previous therapy did not find a significant association. An interesting result, although not significant, was the association between NLR and chronic pelvic pain (OR = 1.9). In the sub-group of patients without previous therapy this association is even stronger (OR = 4.8, 95% CI 0.5–50.2, p = 0.190). The link between NLR and chronic pelvic pain can provide a further hint to the clinician even when taking symptoms into account to develop a particular therapeutic treatment related to the various expressions of NLR. Finally, NLR may enable the creation of customized follow-up protocols that divide patients into high- and low-risk categories for endometriosis recurrence.

## Introduction

Endometriosis is a common, benign, chronic inflammatory condition, with prevalence rates between 6 and 10%. It is typical of reproductive-aged women, 10% of which are affected, but its prevalence increases up to 20–50% in infertile women^1,2^. It is defined as the presence of endometrial gland and stroma outside the uterine cavity and can cause infertility and pelvic pain, that can have a significant negative influence on a patient's quality of life^3,4^.

Endometrial cell attachment outside the uterus is a crucial phase for the development, persistence and progression of disease, through a variety of altered pathways: immunological function (changes of local and systemic activated chemokine), apoptosis, invasion capability, and angiogenesis, caused by proinflammatory, angiogenic and angiostatic chemokines that modulate the severity of endometriosis associated symptoms. In particular, in endometriosis-affected women, the peritoneal fluid is made of elevated levels of activated macrophages and significant variations in the cytokine/chemokine profile; in particular it is noticeably rich in prostaglandins, and these mediators probably play a crucial role in the pathogenesis of the condition as well as in the clinical consequences and symptoms. Changes in local and systemic activated chemokine patterns are what lead to chronic pelvic discomfort in endometriotic patients^5,6^. Additionally, hormonal changes may affect endometrial cells' capacity to multiply, forming and sustaining implants. The overall effect of this variable expression profile between the increased expression of the aromatase enzyme and the decreased expression of 17-hydroxysteroid dehydrogenase type 2 (17-HSD) is a significant rise in the concentration of locally accessible estradiol. Prostaglandin E2 is produced in response to estradiol stimulation, which, in turn, increases aromatase activity^7^.

It has been demonstrated that some cytokines, such as tumor necrosis factor-alpha (TNF-a), interleukin-6 (IL-6), and IL-8, are different in endometriosis-affected women compared to controls^8–10^. For this reason, nowadays numerous researches have concentrated on markers of inflammation in an effort to develop less intrusive approaches for the diagnosis of endometriosis^8–10^. The most extensively studied marker is CA 125 (carbohydrate antigen 125), positive levels of which have been observed in serum, menstrual effluent, and in the peritoneal fluid of this group of women. Although CA 125 is often elevated in advanced endometriosis, the low sensitivity of this diagnostic assay limits its usefulness for detecting minimal and mild disease (stage I and II)^11,12^. On the other hand, a previous meta-analysis have underlined that serum CA 125 had a limited ability to diagnose endometriosis^13^.

It is widely known that systemic inflammatory reactions cause an increase in the neutrophil-to-lymphocyte ratio (NLR). Literature evidence suggest that the NLR may be a predictive indicator for several diseases as well as a measure of the systemic inflammatory response^14–16^, which causes changes in the relative levels of circulating white blood cells; neutrophilia is accompanied by a relative lymphocytopenia^17^. Additionally, it was discovered that women with advanced stage endometriosis had mean NLR values that were considerably greater than those of patients with benign tumors or healthy individuals^18^. Given the aforementioned results, the NLR may rise proportionally as endometriosis progresses since stage-dependent alterations are predicted if a particular biomarker is implicated in the pathophysiology of the condition.

The aim of the study is to evaluate the role of NLR in the real life setting of patients affected by endometriosis in order to evaluate the possible correlation between this biological marker, endometriosis related symptoms, and clinical stage of the disease.

## Material and methods

We performed a retrospective analysis of 199 enrolled premenopausal women affected by endometriosis, who underwent a gynecological examination for chronic pelvic pain at the Department of Obstetrics and Gynecology, IRCCS Fondazione Policlinico San Matteo, of Pavia, from January 2013 to December 2020. Among these patients, 73 received elective surgery with subsequent pathological evaluation and histologically confirmed endometriosis.

Approval from the ethical committee of the IRCCS Fondazione Policlinico San Matteo hospital was obtained. All the patients signed informed consent. The appropriate norms and regulations were followed when performing all methods and procedures.

Data collection included: age, number of pregnancies, comorbidities, smoking status, previous hormonal therapy (either a combined oral contraceptive, progestin only, or GnRH analogue) and their route of administration, duration of therapy, positivity of a recent CA125 test, visual analogue scales for dysmenorrhea and chronic pelvic pain, WBC count with differentials, and AFS disease.

Using the American Society of Reproductive Medicine's (ASRM) updated classification, the amount of endometriosis was evaluated^19^, and the severity of the disease was classified as minimal-to-mild disease (stages I and II) or moderate-to-severe disease (stages III and IV).

The presence of symptoms related to deep endometriosis (pelvic chronic pain and dysmenorrhea), was recorded during pre-surgical gynecological evaluation. Patients underwent a regular preoperative evaluation that included a transvaginal ultrasound and a pelvic examination. Surgery was performed during the follicular phase in all patients.

Differential WBC counts and CA 125 values in serum taken the day of surgery were documented for all trial participants. Five milliliters of peripheral venous blood were used to assay the CA 125, whose detection was performed in the follicular phase. The NLR was defined as the absolute neutrophil count divided by the absolute lymphocyte count (expressed as absolute value 10^3/ml).

Data on sociodemographic and medical conditions were gathered through symptom-focused interviews, in order to assess the presence and severity of any pain symptoms that might be connected to the diagnosis. Dysmenorrhea and chronic pelvic pain, were rated using 10 items on the VAS scale, where 0 represented no pain and 10 represented unbearable pain.

### Statistics analysis

Qualitative variables were described as counts and percentages of each category. Quantitative variables were summarized as mean and standard deviation (SD) or median and interquartile range (IQR). NLR was categorized according to its median value (> 2.62 vs. ≤ 2.62). The association between qualitative variables was assessed by Fisher’s exact test. The comparison of quantitative variables between two groups was evaluated via Mann–Whitney test.

The association between NLR and endometriosis variables (CA 125 > 35 ml/l, moderate-to-severe endometriosis, presence of dysmenorrhea and chronic pelvic pain), adjusting for previous therapy, was evaluated with bivariable logistic regression models. Results from these models are reported in terms of odds ratio (OR), with its 95% confidence interval (95% CI) and p-value.

Two tailed type I error was set at 5%. All the analyses were carried out with Stata 18.0 (StataCorp., College Station, TX, USA).

## Results

Table 1 reports the demographic and clinical characteristics of patients enrolled in the analysis. The overall mean age of the participants was 38 ± 7 years, ranging from 26 to 51. 15 women (20.5%) had a history of smoking. Previous pregnancy was reported in 43.8% of subjects. According to the American fertility society staging system, 11 patients were stage I (15.3%), 17 were stage II (minimal to moderate disease 38.9%), 19 were stage III and the remaining 25 were stage IV (moderate to severe disease 61.1%). 6 patients (8.2%) had a positive family history for endometriosis. 38 patients (52%) were assuming a hormonal therapy, and 2 patients (2.7%) GnRH analogue therapy; median duration of therapy was 3 months. Chronic pelvic pain was reported in 35.6% of women, with a median VAS of 5 among them, dysmenorrhea was reported in 32 (44%) of subjects, with a median VAS of 6.

**Table 1:** Demographic and clinical characteristics of patients.

Variable	Patients N (%)
Age (years), mean ± SD	38 ± 7
Smoking yes, n (%)	15 (20.5)
Pregnancies yes, n (%)	32 (43.8)
Endometriosis stage, n (%)	
I	11 (15.3)
II	17 (23.6)
III	19 (26.4)
IV	25 (34.7)
Previous medical therapy, n (%)	
Estroprogestins	14 (19.2)
POP	24 (32.9)
GnRH analogue	2 (2.7)
No therapy	33 (45.2)
Time of therapy (months), median (IQR)	3 (0–8)
Neutrophiles (cells × 10^3^/ul), mean ± SD	6.1 ± 4.5
Lymphocytes (cells × 10^3^/ul), mean ± SD	1.8 ± 0.7
VAS Chronic pelvic pain in all patients, median (IQR)	0 (0–5)
Chronic pelvic pain Yes, n (%)	26 (35.6)
VAS Chronic pelvic pain, median (IQR)	5 (3–7)
VAS Dysmenorrhea in all patients, median (IQR)	0 (0–6)
Dysmenorrhea Yes, n (%)	32 (43.8)
VAS dysmenorrhea, median (IQR)	6 (5–8)
CA125 (ml/l), n (%)	
> = 35	17 (23.3)
< 35	56 (76.7)

Analyzing the neutrophiles, the mean ± SD value was 6.1 ± 4.5 × 10^3^/ul, while for lymphocytes mean ± SD value was 1.8 ± 0.7.

Table 2 describes the association between NLR values and clinical characteristics. The analysis did not delineate a significant association between these variables and NLR.

**Table 2:** Association between NLR value (≤ 2.62 and > 2.62) and clinical characteristics.

Variable	NRL ≤ 2.62 (N = 37)	NLR > 2.62 (N = 36)	p-value
CA125 > = 35 ml/l, n (%)	11 (29.7)	6 (16.7)	0.269
Stage III–IV, n (%)	23 (62.2)	21 (60.0)	> 0.90
VAS Chronic pelvic pain, median (IQR)	0 (0–2)	0 (0–5)	0.274
VAS Dysmenorrhea, median (IQR)	0 (0–6)	0 (0–5)	0.710

**Figure 1:**
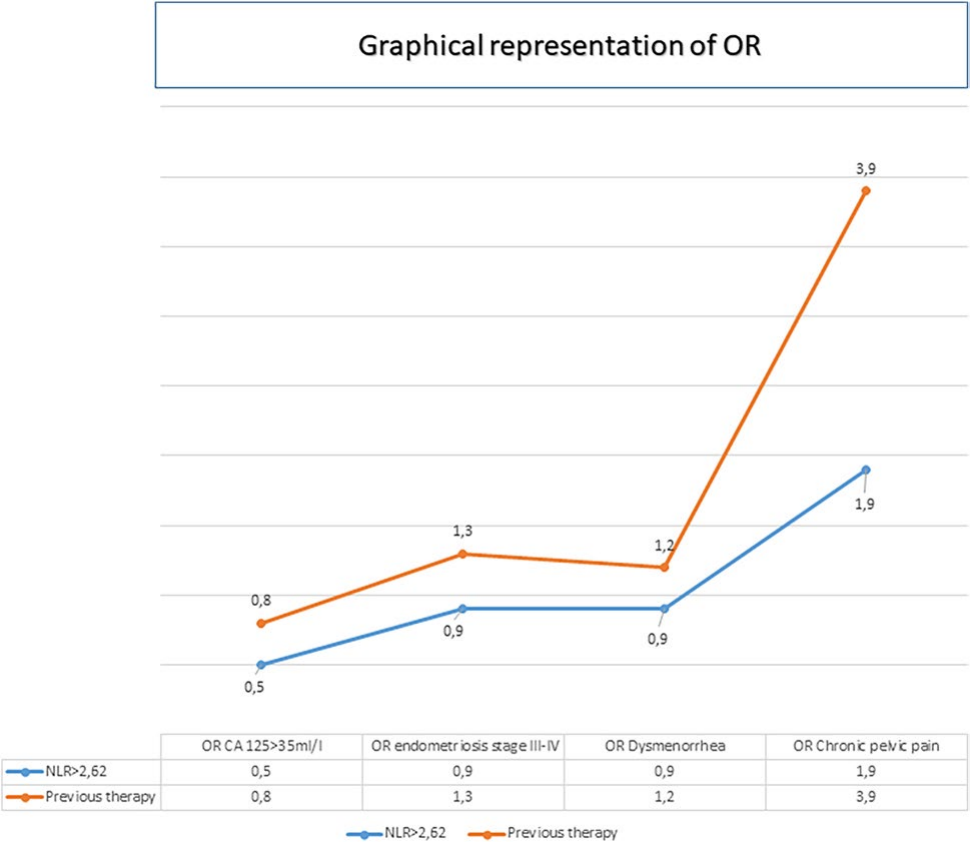
Garphical rapresentation of OR between NLR values and CA 125, endometriosis stage, presence of dysmenorrhea and presence of chronic pelvic pain, adjusting for previous therapy.

Table 3 and Fig. 1 show the comparison between NLR values and CA 125, endometriosis stage, presence of dysmenorrhea and presence of chronic pelvic pain, adjusting for previous therapy (bivariable models). This model failed to demonstrate any correlation between NLR and CA125, endometriosis stage and dysmenorrhea. An interesting result, although not significant, was the association between NLR and chronic pelvic pain (OR = 1.9). In the sub-group of patients without previous therapy this association is even stronger (OR = 4.8, 95% CI 0.5–50.2, p = 0.190).

**Table 3:** Logistic bivariable models between CA 125, VAS of dysmenorrhea, chronic pelvic pain and NLR.

	CA125 > = 35 ml/l		Endometriosis stage III-IV		Dysmenorrhea present		Chronic pelvic pain present	
	OR (95% CI)	p-value	OR (95% CI)	p-value	OR (95% CI)	p-value	OR (95% CI)	p-value
NLR								
< = 2.62	Ref		Ref		Ref		Ref	
> 2.62	0.5 (0.2–1.4)	0.186	0.9 (0.4–2.4)	0.858	0.9 (0.3–2.1)	0.724	1.9 (0.7–5.1)	0.232
Previous therapy								
No	Ref		Ref		Ref		Ref	
Yes	0.8 (0.3–2.4)	0.641	1.3 (0.5–3.3)	0.658	1.2 (0.5–3.2)	0.693	3.9 (1.2–12.3)	0.020

## Discussion

Although NLR was not statistically associated with any outcome, a trend toward a high probability of chronic pelvic pain in patients with higher NLR was observed. There are several possible reasons for the association between NLR and chronic pelvic pain which could be traced back to the immunological alterations that result from the pathogenesis of endometriosis itself. In fact, NLR is also a known, yet unspecific, marker for chronic inflammation and this may contribute to its elevation on the setting of endometriosis^18,20^.

CA 125 as well, has been found to be elevated also in the menstrual effluent, the peritoneal fluid and the serum of endometriotic patients. Despite the fact that it is often positive in advanced endometriosis, its lack of an adequate sensitivity and specificity fails to make it a suitable diagnostic marker^21^.

There are also other known hematological effects of endometriosis, namely a neutrophilia, lymphopenia (contributing to the increase in NLR), decrease in hemoglobin, platelets, eosinophils, and basophils^20^.

The findings of the study are coherent with previous ones that reported, in endometriotic patients, a decreased proliferation of peripheral blood lymphocytes, resulting in a mild lymphopenia, in response to the recognition of endometrial cells and antigens^22^.

The lack of association between NLR, CA 125 and the medical therapy is likely due to the highly variability in treatment duration among the patients recruited in the study; in fact, some of them were undergoing hormonal therapy for decades. Perhaps a stronger association could have been detected in the first months after initiation of pharmacological management. Other confounding factors are the different types of medical therapies: different estrogens and progestins (use of combined pills or progesterone only pills), use of different treatment schemes, namely continuous administration, regular use or long cycle, the use of intramuscular injections of GnRH analogue triptorelin, and, eventually, the use of local routes of administrations in the form of intrauterine devices, vaginal rings and transcutaneous patches.

Contrary to previous studies, that have found a correlation between endometriosis and preoperative CA 125, like the one by Yavuzcan A, et al.^23^ we did not find a similar association. An interesting aspect of past research on the topic of the associations between NLR and endometriosis is that Kim SK, et al.^24^ found a positive correlation with the size of endometriomas.

It is currently unknown how predictive NLR is for determining the likelihood of developing symptoms associated with endometriosis. Furthermore, there isn't a set baseline value for NLR, and it's likely influenced by a number of clinical conditions, such as immunodepression, steroid medication, stressful conditions, as well as patient behavior, for example smoking. Nevertheless, NLR appears as a useful prognostic indicator, and cost-effective and non-invasive biomarker of endometriosis, especially regarding the correlation with pelvic pain. There is compelling evidence that laboratory biomarkers that are readily available in standard clinical practice, such as in endometriosis, can have a role in the prediction of outcomes for a range of conditions, not just malignancies. We achieved this by using a straightforward, repeatable, and reasonably priced clinical tool that could be ideally implemented in clinical practice across all contexts. Moreover, these characteristics could be most effectively and favorably applied to early-stage endometriosis, for which conservative treatments are increasingly being used as the first line. Beyond the typical predictive value acquired by clinical-pathological and biomolecular markers, this simple parameter may offer extra value.

In addition, preoperative NLR values may be a useful prognostic biomarker, particularly in younger patients, where deep endometriosis may have a detrimental effect on future fertility and pregnancy. Finally, identifying patients at higher risk of infiltrating endometriosis will enable the creation of tailored diagnostic and therapeutical planning for patients distinguishing, thus, between those who are at high and those at low risk of persistence or recurrence of the disease. In other words, our data suggested that NLR is a relevant clinical option to guide the choice of treatment, based on patient’s symptoms and on the value of the NRL itself.

The main limitation of the study is the absence of a control group, which can represent a confounding factor for the interpretation of the results. Other weaknesses are the design, being a monocentric retrospective study, and the lack of an appropriate NLR follow up, which could further clarify the results. The main strength is that the results provide solid evidence of the association between NLR and chronic pelvic pain, that opens up several clinical opportunities to improve the standard of care for the patients. In fact, it can be used as a device to objectify the subjective pain sensation reported, and this may hopefully reduce the diagnostic delay. In the future this could become a tool allowing therapy monitoring and therefore helping in the decision-making process for intensification of medical care. Finally, it paves the way for further research on the topic.

## Conclusion

The link between NLR and chronic pelvic pain can provide a further hint to the clinician even when taking symptoms into account to develop a particular therapeutic treatment related to the various expressions of NLR. The role of neutrophil/lymphocyte ratio in clinical practice can be an objective, measurable proxy of chronic pelvic pain thus being a valuable tool in the diagnosis of endometriosis, with the possibility of decreasing the diagnostic delay, and as a precious aid in the clinical decision-making process and treatment option. Finally, NLR may enable the creation of customized follow-up protocols that divide patients into high- and low-risk categories for endometriosis recurrence. On the contrary, the value of this index as a prognostic device is still unclear, therefore more research is needed on this topic.

## Data Availability

Correspondence and requests for materials should be addressed to M.D.
